# Speculations on the Role of Successful Intelligence in Solving Contemporary World Problems †

**DOI:** 10.3390/jintelligence6010004

**Published:** 2018-01-23

**Authors:** Robert J. Sternberg

**Affiliations:** Department of Human Development, College of Human Ecology, Cornell University, B44 MVR, Ithaca, NY 14853, USA; rjs487@cornell.edu

**Keywords:** intelligence, creativity, wisdom

## Abstract

In this article, I argue that conventional views of intelligence and its measurement have contributed toward at least some of the societal problems of today. I suggest that to escape from a degenerative process, society needs to consider the importance not only of intelligence, as conventionally defined but also of successful intelligence, involving in addition to conventional analytical intelligence, common sense, creativity, and wisdom.

## 1. Introduction to the Symposium on the Application of Theory and Research on Intellectual Abilities to the Solution of Vexing World Problems

This article is the first in a symposium of thought pieces on the following topic: “If intelligence is truly important to real-world adaptation and IQs have risen 30+ points in the past century (Flynn effect), then why are there so many unresolved and dramatic problems in the world and what can be done about it?”

The goal of the symposium is to have articles representing a number of different points of view with regard to constructs that might be relevant to solving serious problems facing the world today. These articles (including the present one) are speculative. We really do not have validated scientific data on how, say, analytical intelligence, creativity, common sense, or wisdom can be applied effectively to problems of climate change, poverty, or pollution. But if psychological scientists shy away from these problems, they may leave all of the speculation to individuals who bring no scientific perspective at all to these issues. Authors in the symposium are not expected to provide solutions but rather to point the way for how various constructs might help policy-makers and others address world problems.

My motivation in organizing this symposium is that I believe that psychology and related disciplines have a great deal to contribute to the solution of world problems but have been sidelined in favor of other fields, such as economics and the law. In this symposium, we examine in particular the role of theory and research on intellectual abilities. Examples of some of the constructs to be considered are intelligence (as conventionally defined), rational thinking, creativity, reasoning (biased and unbiased) and wisdom. These are all components of successful intelligence.

In particular, I have chosen invitees whom I view as experts on constructs particularly relevant to the solution of world problems and who I believe have an interest in the solution of such problems. Again, the articles will provide no final answers or detailed solutions but rather, directions for theory, research and policy analysis, as appropriate. Obviously, articles for this symposium involve some dose of personal opinion and speculation but all are to be grounded in psychological theory and research. 

So, here is the first article, on successful intelligence. Successful intelligence is one’s ability to formulate, execute, evaluate and then, as needed, reformulate one’s plans for one’s life [1,2]. It encompasses creative, analytical, practical and wise thinking. That is, one needs creative thinking to generate ideas, analytical thinking to decide whether the ideas are good ideas, practical thinking to implement the ideas and persuade others of their value and wise thinking to ensure that the ideas help to achieve a common good, in the long- as well as the short-term.

## 2. A Mesopotamian Tale

There is a Mesopotamian tale, retold by W. Somerset Maugham, about a servant who, seeing Death staring at him in a strange way in Baghdad, flees to Samarra to escape Death. When the merchant for whom the servant works sees Death in Baghdad, he asks Death why he gave the man such a strange stare. Death explains that the stare was only because he was surprised to see the servant in Baghdad, when in fact Death had an appointment with the servant the next day in Samarra.

Are we, in the world, creating a race to Samarra? I suggest that viewing intelligence in a conventional way (see essays in [3,4]) metaphorically may be leading us toward, rather than away from, catastrophe in Samarra. Rather, we need to view intelligence in terms of “successful intelligence,” or the ability to formulate, execute, and evaluate plans for the conduct of a life that is personally meaningful and fulfilling [5]. Particularly important in this formulation, as described below, is wisdom.

## 3. Conventional Intelligence Is Not Enough to Solve World Problems

What, exactly, is intelligence (see essays in [4])? Intelligence often is seen in terms of ability to learn and reason and in terms of adaptation to the environment [6,7,8,9]. Traditionally, it has been operationalized in terms of what IQ tests or tests of abstract thinking measure and of the processes underlying such tests (see [6,10]).

I have suggested that the processes of intelligence can be understood in terms of three kinds of components of intelligence [11,12]. *Metacomponents* are higher order executive processes that plan what to do, monitor it while it is being done, and evaluate it after it is done. The main metacomponents are:

- Recognizing the existence of a problem (e.g., that intelligence tests may favor students from some cultural backgrounds over students from other cultural backgrounds);
- Defining the nature of the problem (e.g., that the students who are disfavored may actually be quite intelligent in their natural environmental contexts, even if not in the context of traditional intelligence tests);
- Constructing a mental representation of the problem (e.g., learning what kinds of tasks are representative of adaptive and hence intelligent performance in various cultural settings);
- Formulating a strategy to solve the problem (e.g., planning to devise tests relevant to various cultural milieus);
- Monitoring problem solving while it is in process (e.g., empirically determining whether the tests that have been created indeed are relevant to the various milieus);
- Evaluating problem solving after it is completed (e.g., determining whether the new tests have construct validity in the environments for which they are intended) (see [13,14] for related ideas regarding creativity).

Performance components actually solve the problems. And knowledge-acquisition components learn how to solve the problems in the first place [11]. Other scholars’ theories have similar problem-solving processes (see, e.g., [15]).

There are broader views of abilities (e.g., [5,16]) but society has yet to adopt them. Even with respect to traditional views, intelligence has proven to be somewhat problematical in terms of its role in society (see, e.g., [17]).

Professor James Flynn of the University of Otago has found that during the 20th century, IQs rose worldwide about 3 points per decade, or roughly 30 points [18]. Even better, in the United States, IQs are continuing to rise [19]. A difference of 30 points is huge: It is the difference between a gifted IQ and an average one and between an average IQ and one at the borderline of labeling someone “intellectually challenged.” Average IQs remain 100 because test publishers periodically re-standardize tests to make the average 100. Without doubt, increased levels of intelligence in a traditional sense have brought societies in the world many blessings, such as advances in science and technology.

Unfortunately, the steep rise in IQ has bought us, as a society, much less than anyone had any right to hope for. People are probably better at figuring out complex cell phones and other technological innovations than they would have been at the turn of the twentieth century. But in terms of our behavior as a society, are you impressed with what 30 points has brought us? Higher IQs have not brought with them generally satisfying solutions to some of the world’s or the country’s major problems—rising income disparities, climate change, pollution, organized violence, terrorism, deaths by opioid poisoning, among others. Today many children are being poorly educated and many still do not learn to read well [20]. Depending on one’s opinions, one may see many other serious problems as well but it is not clear how many, if any of those have been solved.

What all these problems have in common is that the use of conventional intelligence to maximize gains for one group or another can result in a reduction of the common good. Conventional intelligence easily can be used to maximize the gains of an individual or a group at the expense of other individuals or groups. Policies that benefit particular groups in the short term but that hurt the common good in the long term also then end up hurting in the long term the groups the received the short-term benefits. For example, if climate change continues, eventually everyone will suffer, regardless of short-term benefits to particular groups.

Most of the academic tests used in schools—in the United States, SATs, ACTs, GREs and so on; in other countries, similar tests with different names—are basically IQ proxies. They are not the same as IQ tests but scores on them are moderately to highly correlated with IQ [21,22,23]. Our society, in placing so much emphasis on scores on standardized tests, is making a serious and possibly irreversible mistake [11,12,24,25,26]. In my view, we are creating an educational race that rewards people who score highly on skills that will help their own life chances to a small to moderate extent [25,26,27,28]. But the race does little to choose winners who will create a positive, meaningful, and enduring difference to our future [29,30]. We have created a race to Samarra. The skills our schools emphasize matter somewhat for school and life success but they are the beginning, not the end of the story of what matters [31,32].

We need to be developing virtues such as good character, compassion, active citizenship and ethical leadership, and other important skills such as creativity, common sense and wisdom—using one’s knowledge and skills for a common good, by understanding other people’s points of view and by ethically balancing one’s own interests with other people’s and larger interests of society and the world [31,33,34]. Judging by international trends, we seem to be doing much the opposite. “Other people’s interests” seem to extend largely to people we imagine to be like us but not much to those in our self-constructed outgroups. Much of the current (2018) political leadership around the world nicely illustrates this idea of favoring people perceived to be like the leaders (e.g., people of a certain economic group, religion, socially-defined race, ethnicity, or political persuasion).

Conventional intelligence is insufficient for creating a better world. Moreover, it has a dark side [33,34,35]. The dark side is rather obvious: Intelligence can be used for good ends (e.g., Nelson Mandela, Martin Luther King) but it equally well can be used for bad ends (e.g., Adolph Hitler, Josef Stalin, and those, like the fictional Lord Voldemort, who must not be named). Intelligence is a measure of adaptive skills [36,37,38]. But those skills are for oneself, not for society or the world as a whole. People can adapt just fine but sometimes, at the expense of others. Many problems in the world show how some people are learning to adapt just fine and are just as fine with leaving others behind. 

Intelligence is thus important but provides no guarantee of a better world. Intelligence was used to develop nuclear weapons, poisonous gases, and the fossil fuels in part behind climate change: Intelligence can make the world much better but it also can destroy the world, at least as we know it.

## 4. Creativity Is Not Enough to Solve World Problems

Creativity is partly an attitude toward life, not merely an ability [39,40]. Moreover, it always takes place within a system [41,42,43,44,45,46,47]. Creativity can provide part of the answer to creating a better world [48,49]. The creative attitude is one of buying low and selling high—taking good ideas that others are reluctant to accept, persuading others of their value, and then moving on to the next unpopular idea [48]. Most people fail to be creative not because they cannot be but rather because they are afraid to be. Creativity involves three kinds of “defiance” [50]:

- **Defying the crowd:** Creative people are willing to stand up to the resistance that creativity almost inevitably sparks in others (the crowd). Often people, including scientists, value creativity except when it threatens them personally—they prefer ideas that do not require them to challenge their fundamental ways of thinking.
- **Defying oneself:** Creative people are willing to stand up to their own prior ideas and conceptions and to move on as they change, the world changes and their potential contribution to the world changes. Often people are willing to stand up to others but not to themselves, with the result that their “new” ideas are minor re-workings of their old ideas—“old wine in new bottles.”
- **Defying the Zeitgeist:** Creative people are willing to stand up to the often preconscious presuppositions under which they and the crowd have operated. An example has been significance testing: For a long time, the use of significance testing has been simply part of the Zeitgeist, whereas now Bayesian researchers more and more are challenging it. Often people do not want to challenge the presuppositions with which they have become comfortable and that define their personal and professional lives. Creative people do so.

Creativity includes insightful thinking [51,52] and other kinds of innovative thought. There are multiple kinds of creativity [45,49], ranging from baby steps (what Kaufman & Beghetto refer to as “little c”) forward to giant steps that change the face of the world (what Kaufman & Beghetto refer to as “Big C”). Creativity can be measured and developed, at least to some extent, in any part of the world [14]. The computer on which this article is being written and most of the conveniences of modern life, are possible only because of creativity, or the creation of new ideas and products that are both novel and somehow useful [53]. But creativity also has a dark side [54,55]. People can use creativity for good purposes (e.g., formulation of medicines to cure diseases, composition of symphonies, writing of classic novels) or for bad purposes (e.g., designing devastating bombs, formulating and executing terrorist attacks, designing novel means for committing genocide and perhaps for covering up the acts). The world needs more than just creativity, just as it needs more than just intelligence.

## 5. Common Sense

Practical intelligence, or “common sense,” is involved when skills are utilized, implemented, applied, or put into practice in real-world contexts. It involves individuals applying their abilities to the kinds of problems they confront in daily life, such as on the job or in the home. Practical intelligence involves applying the components of intelligence to experience so as to (a) adapt to, (b) shape, and (c) select environments. Adaptation is involved when one changes oneself to suit the environment. Shaping is involved when one changes the environment to suit oneself. And selection is involved when one decides to seek out another environment that is a better match to one’s needs, abilities, and desires. People differ in their balance of adaptation, shaping, and selection and in the competence with which they balance among these three possible courses of action. One would hope that world leaders would apply these skills with facility. They often do not.

Underlying practical intelligence is tacit knowledge [56]. The concept of tacit knowledge reflects the idea that much of the knowledge relevant to real-world performance is acquired through everyday experiences without conscious intent. Tacit knowledge guides action without being easily articulated. It is knowledge needed in order to work effectively in an environment that one is not explicitly taught and that often is not even verbalized [25,27]. My colleagues and I represent tacit knowledge in the form of production systems, or sequences of “if-then” statements that describe procedures one follows in various kinds of everyday situations [27].

We have studied tacit knowledge in domains as diverse as bank management, sales, academic psychology, primary education, clerical work, social interaction and military leadership [27,57]. The measurement of tacit knowledge derives from an assessment of how individuals rate responses to practical problems. The format on tacit-knowledge tests is akin to that on situational-judgment tests. Individuals are presented with a brief problem description and are asked to evaluate the quality of potential solutions to the problem. For example, in a hypothetical situation presented to a business manager, a subordinate whom the manager does not know well has come to him for advice on how to succeed in business. The manager is asked to rate each of several responses (usually on a 1 = low to 9 = high scale) according to its importance for succeeding in the company. Examples of responses might include (a) setting priorities that reflect the importance of each task; (b) trying always to work on what you are in the mood to do; and (c) doing routine tasks early in the day to make sure you get them done. Tacit-knowledge (TK) tests typically consist of several problem situations. Responses are scored by comparing an individual’s ratings on all the alternatives to a standard based on expert or consensus judgment.

The score an individual receives on a TK test is viewed as an indicator of his or her practical ability. Previous research has examined the relationship of TK scores to domain-specific experience, general cognitive ability and various indicators of performance. Generally, individuals with greater experience in a domain (e.g., business managers versus business students) receive higher TK scores. Tacit-knowledge scores also correlate fairly consistently with performance across a variety of domains. Individuals with higher TK scores have been found to have higher salaries, better performance ratings, more productivity and to work in more prestigious institutions [58].

Finally, TK tests appear to tap abilities that are distinct from those measured by traditional intelligence or ability tests. The correlations between scores on TK tests and scores on traditional intelligence tests have ranged from negative to moderately positive [27]. More importantly, TK scores have been found to explain performance above and beyond that accounted for by tests of general cognitive ability [27]. Thus, TK tests offer a promising approach for assessing an individual’s practical abilities.

Given that TK tests show minimal correlations with IQ, it might make sense to use such tests for selecting individuals who will be in a position to affect policy, at whatever level. Our current system of selection is such that someone with a high IQ but minimal common sense easily could end up in such a position. Come to think of it, we almost certainly have in the world myriad such people—people affecting important world decisions who were chosen for their academic intelligence but not for their common sense (see also [59]).

## 6. We Also Need Wisdom to Solve World Problems

Wisdom in conjunction with creativity, common sense, and conventional intelligence, can help to create a better world [60,61,62]. I believe wisdom should be seen as an integral part of successful intelligence, to the extent that intelligence encompasses the idea not only of success for the individual but also for society and the world. There are many definitions of wisdom but almost all of them point to wisdom as a key to creating a better world. In my own definition [61], wisdom involves using both one’s analytical and practical intelligence and one’s creativity, as well as one’s knowledge base, for a common good over the long as well as the short term. So, at least by definition, wisdom cannot be used toward dark ends. One achieves the common good by balancing one’s own interests with the interests of others and larger interests (such as of one’s family, one’s community, one’s nation, or the world), over the long as well as the short term, through the infusion of positive ethical values. However one defines wisdom, it is clearly a key to solving world problems. 

### 6.1. Kinds of Wisdom

Wisdom can be seen as being of different kinds, depending on two dimensions, domain generality and depth (see Table 1).

*Domain-general deep wisdom* is the kind of wisdom we often think of when we think about wisdom. It refers to people who reflect deeply on complex problems across a wide variety of domains and then reach wise solutions. The thinking of great thinkers such as Socrates or Aristotle would fall into this category but so would the thinking of great recent leaders such as Martin Luther King or Nelson Mandela.

*Domain-general shallow wisdom* is the kind of wisdom we may think of when we watch a Hollywood movie in which a person, usually an elderly person, gives wise advice, especially to a younger person. The person is wise enough for a movie or a TV show but the level of advice is at the level of the old TV show, “Father Knows Best.” The individual exhibits wisdom, perhaps across a number of domains but for simple everyday problems that perhaps younger people (and some older ones) just have not yet learned how to solve. This is the kind of wisdom we often impart to our children in the face of the everyday problems they face at school.

*Domain-specific deep wisdom* applies to deep thinking about complex problems but within a relatively narrow domain. Someone, for example, may be wise in giving career advice but terrible in giving personal advice, or vice versa. Some of us have had wise mentors, for example, who gave us sound professional advice but who made a mess of their personal lives and perhaps the personal lives of others as well. 

*Domain-specific shallow wisdom* is superficial wisdom that applies simply in a single domain. There is a lot of that around in the political sphere. 

### 6.2. Non-Wisdom

Regrettably, much more common in the world than wisdom is non-wisdom, or the lack of wisdom (see Table 2).

*Quasi-wisdom* is near-wisdom, or advice that comes with incomplete reflection or insight. A person may give advice that cosmetically seems wise but that misses important factors that should have been taken into consideration.

The *veneer of wisdom* occurs when someone, perhaps someone in a position of leadership or authority, is thought to be wise by virtue of his or her position, or perhaps by virtue of other kinds of skills, such as intelligence or creativity, or by virtue of a vast store of knowledge. It is a mere appearance of wisdom, or perhaps wisdom that is a “micron thick.” The person may be smart or creative or knowledgeable but be unable or unwilling to use his or her skills or knowledge wisely—toward a common good.

*Pseudo-wisdom* is the deliberate attempt to appear wise by someone who is anything but, for example, a leader who uses his or her position of authority to serve his or her personal wants and desires or the wants and desires of his or her family or perceived allies, while trying to convey the impression of seeking a common good.

*Dark pseudo-wisdom* is like pseudo-wisdom, except that it is the appearance of wisdom in the service of evil ends, such as abusing children or encouraging people to become suicide bombers. False religious leaders may take advantage of their position to harm others rather than to help them.

### 6.3. Foolishness

Whereas non-wisdom is the lack or nullity of wisdom, the opposite of wisdom is foolishness [63,64] (Table 3). If lack of wisdom is like a “zero,” foolishness is like a “negative number.” People can be highly intelligent or even creative and yet foolish. Indeed, high intelligence can be a risk factor for foolishness, precisely because people who are highly intelligent may believe they are immune to foolishness. Foolishness is exhibited through a series of six cognitive fallacies [64]. First, the *unrealistic-optimism fallacy* occurs when people think they are so smart and effective that they can do whatever they want. Second, the *egocentrism fallacy* occurs when people start to think that they are the only ones that matter, not the people who rely on them. Third, the *omniscience fallacy* occurs when people think that they know everything and lose sight of the limitations of their own knowledge. Fourth, the *omnipotence fallacy* occurs when people think they are all-powerful and can do whatever they want. Fifth, the *invulnerability fallacy* occurs when people think they can get away with anything, because they are too clever to be caught; and they figure that even if they are caught they can get away with what they have done because of who they imagine themselves to be. And finally, sixth, the *ethical disengagement* fallacy occurs when people think that ethics are important—for others but not for themselves. They see themselves as above ethical concerns.

## 7. Conclusions

The world is facing huge, pressing, and even frightening problems—terrorism, climate change, increasing income disparities, drug abuse, a divided society, and feelings of many of hopelessness, especially, in some cases, after people see the leaders their fellow citizens choose or tolerate. Successful intelligence, integrating creative, practical, and analytical aspects of intelligence as well as wisdom, provides the potential for solutions to serious world problems. Successful intelligence viewed broadly draws on creativity, conventional (analytical) intelligence, common sense, and wisdom because it often requires people to come up with novel solutions, to analyze whether the solutions are indeed good ones, to implement the solutions and persuade other people of their value and most of all, to ensure that the solutions help to achieve a common good. The world today needs more creativity and wisdom and less foolishness. Our schools need to teach for wisdom [65] and develop broadly adaptive, creative, street-smart, wise and ethical leaders, not just traditionally smart and politically savvy ones [66]. Many of our societies in the world today have created for their members a race but unfortunately, a race to Samarra. Successful intelligence, broadly defined to include wisdom, is a key to finding a different and better race for the societies of the world to run.

## Figures and Tables

**Table 1:** Kinds of Wisdom.

Domain Generality/Depth of Wisdom	Deep	Shallow
Domain General	Domain-General/Deep Deeply insightful advice across domains	Domain-GeneralShallow Modestly insightful advice across domains
Domain Specific	Domain-Specific/Deep Deeply insightful advice in a single domain	Domain-Specific/Shallow Modestly insightful advice in a single domain

**Table 2:** Kinds of Non-wisdom.

Kinds of Non-Wisdom	Manifestation
Quasi-wisdom	Near wisdom—incomplete reflection or insight
Veneer of wisdom	False appearance of wisdom as a result of position of power or authority
Pseudo-wisdom	False appearance of wisdom motivated by self-interest
Dark pseudo-wisdom	False appearance of wisdom motivated by evil intentions

**Table 3:** Kinds of Foolishness.

Fallacy	Manifestation
Unrealistic optimism	“If it’s my idea, it must be good”
Egocentrism	“It’s all about me”
False omniscience	“I know everything I need to know”
False omnipotence	“I am all-powerful”
False invulnerability	“No one can get back at me”
Ethical Disengagement	“Ethics are important for other people”
